# Drill needle aspiration biopsy for submucosal tumors in an experimental study

**DOI:** 10.1007/s10120-016-0630-4

**Published:** 2016-08-16

**Authors:** Masaya Uesato, Tomohide Tamachi, Naoyuki Hanari, Yorihiko Muto, Akiko Kagaya, Ryuma Urahama, Yukiko Ogura, Hiroshi Suito, Akira Nakano, Mizuho Aikawa, Takashi Oide, Hisahiro Matsubara

**Affiliations:** 1 Department of Frontier Surgery, Chiba University Graduate School of Medicine, 1-8-1 Inohana, Chuo-ku, Chiba, Chiba-shi 260-8670 Japan; 2 Department of Diagnostic Pathology, Chiba University Graduate School of Medicine, Chiba, Japan

**Keywords:** Drill needle, Aspiration, FNA, SMT, GIST

## Abstract

**Background and aims:**

EUS-guided FNA biopsy has been widely performed to aid in the diagnosis of submucosal tumors (SMTs). However, in cases of small tumors, the diagnostic yield of EUS-FNA is poor. Therefore, it is necessary to develop a new needle for the diagnosis. We developed a device with a new mechanism that we refer to as a drill needle aspiration biopsy (DNAB). The aim of this study was to evaluate the use of DNAB in resected gastric SMT specimens.

**Methods:**

A drill needle with a sharp tip and wide ditch was inserted into a catheter for angiography. Continuous suction is enabled through the catheter at the tip. DNAB was performed with one pass and one stroke in 13 gastric SMTs resected by operation. Similarly, FNA was performed by one pass and ten strokes. These gastric tumors included nine diagnosed gastrointestinal stromal tumors and four undiagnosed SMTs by preoperative examinations. The tissue quantity between DNAB and FNA was macroscopically and microscopically examined.

**Results:**

All 13 drill biopsy specimens were obtained. Additionally, all 13 gastric SMTs, including 4 undiagnosed tumors, could be diagnosed by DNAB. The quantity of each specimen obtained by DNAB was macroscopically and microscopically much greater than that by FNA. In particular, for tumors <25 mm in the longer axis, the ratio of microscopic diagnosable cases was 100 % (7/7) for DNAB and 42.9 % (3/7) for FNA.

**Conclusions:**

DNAB is a novel method that can obtain more tissue than FNA for small gastric SMT.

## Introduction

Gastrointestinal stromal tumor (GIST) was first defined in 1983 as a tumor in the gastrointestinal tract and mesentery, characterized by a specific histological and immunohistochemical pattern [1]. Because it is well recognized that all GISTs have some degree of malignant potential, they may need to be resected, even if they present as small localized lesions [2]. Differentiating these lesions from benign submucosal lesions such as leiomyomas or schwannomas is crucial. However, standard endoscopic biopsy specimens are typically nondiagnostic because the mucosa overlying the submucosal tumors (SMTs), including GIST, is normal. In such cases, an endoscopic ultrasound-guided fine-needle aspiration biopsy (EUS-FNA) is considered to be a reliable and accurate method for the evaluation of SMTs. However, when the size of SMTs is small, the diagnostic yield of EUS-FNA is poor [3, 4]. Therefore, it is necessary to develop a new needle for the diagnosis of SMTs. We herein devised a new mechanism, which we refer to as a drill needle aspiration biopsy (DNAB). Our aim was to evaluate the use of DNAB in resected gastric SMT specimens.

## DNAB characteristics and procedure

**Fig. 1:**
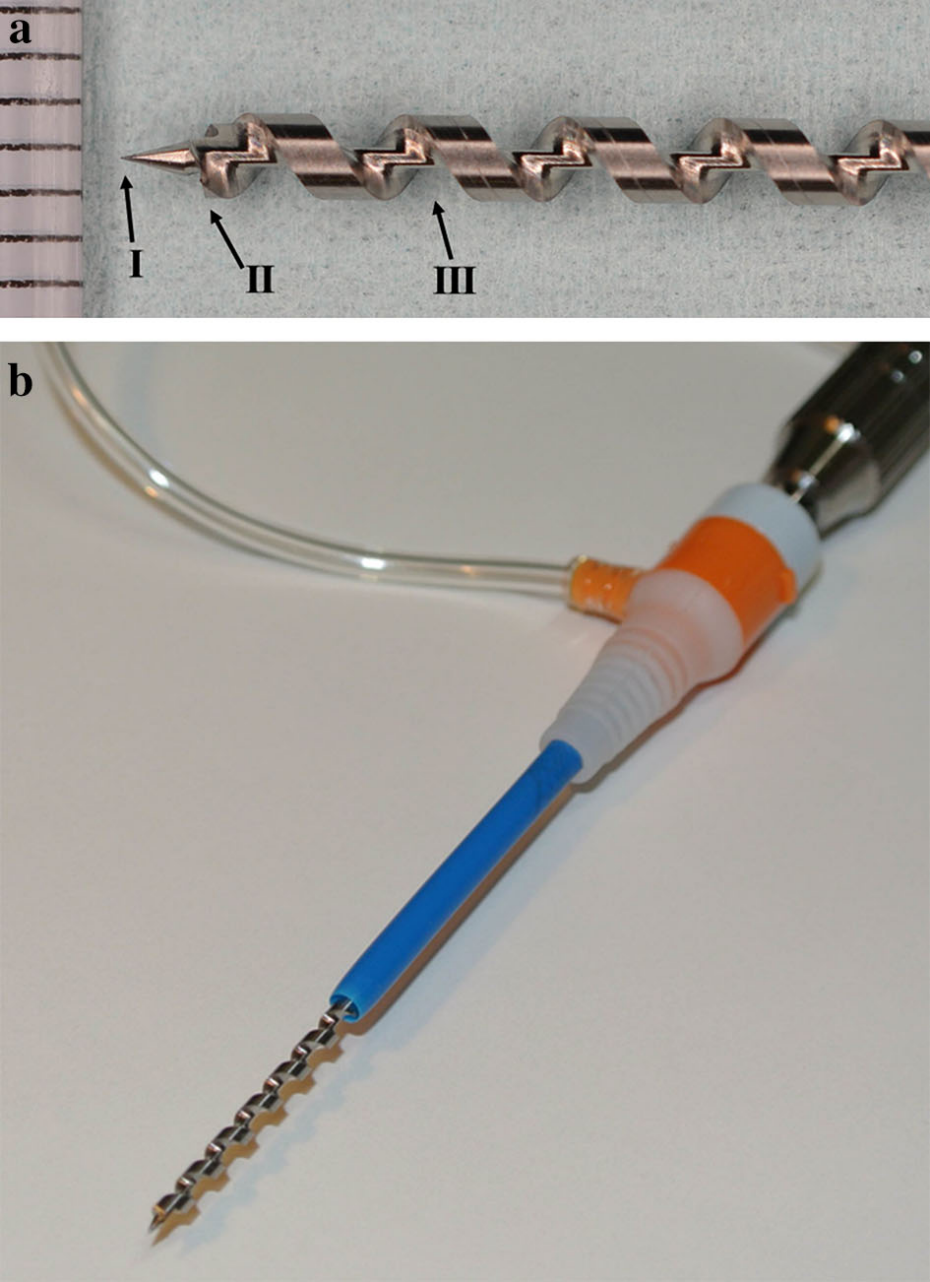
**a** The drill has three main characteristics: a sharp tip (*I*), front-like cutter (*II*) and deep helical ditch (*III*). **b** The diameter of the drill is 2 mm, and the helical ditch is 30 mm. The drill is inserted in a 7-Fr sheath for catheter angiography

**Fig. 2:**
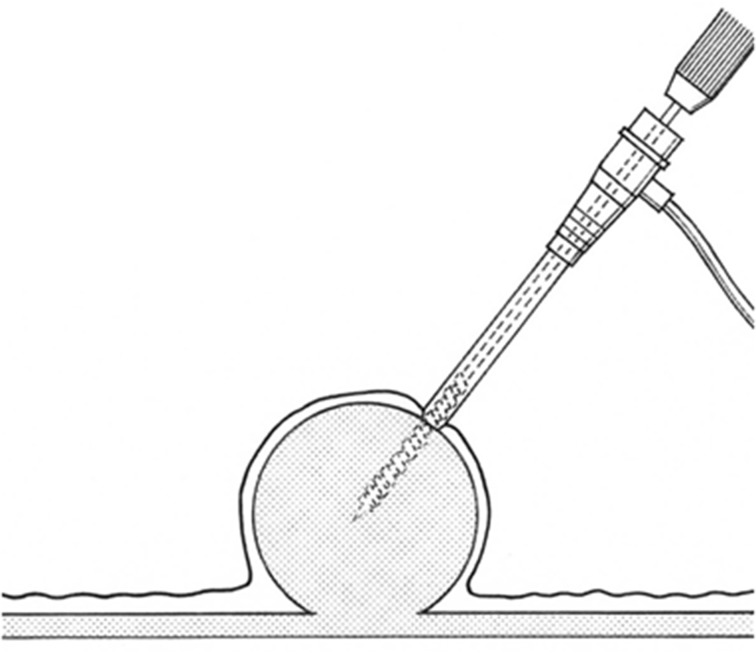
The tip of the sheath adheres to the surface of the SMT. Continuous suction using a 10-ml syringe through the sheath is applied. Then, the drill is manually turned and inserted only once into the SMT

We designed a special drill that was manufactured by TOKO Co., Ltd. (Tokyo, Japan). The drill has three main characteristics: a sharp tip, front-like cutter and deep helical ditch (Fig. 1a). The drill diameter is 2 mm, and the helical ditch is 30 mm. The drill is inserted in a 7-Fr sheath for catheter angiography (Medikit Co., Ltd., Tokyo, Japan) (Fig. 1b). First, the tip of the sheath adheres to the surface of the SMT. Continuous suction using a 10-ml syringe through the sheath is applied. Then, the drill is manually turned and inserted only once into the SMT (Fig. 2). Finally, it is pulled out under turning.

## Materials and methods

We examined a total of 13 resected gastric SMT specimens from consecutive patients (8 males and 5 females) with a mean age of 63.0 years (range 33–78 years) who underwent an operation at the Department of Frontier Surgery, Chiba University Hospital, between March 2013 and July 2015. Among 13 gastric SMTs, 9 were diagnosed GISTs, and 4 were undiagnosed SMTs by preoperative examinations. Thirteen gastric SMTs were fixed on the corkboard, and DNAB was performed under continuous suction. Similarly, FNA with a 22-gauge needle (ExpectTM, Boston Scientific, Marlborough, MA) was performed using one pass and ten strokes at a site different from the insertion site of DNAB in SMT. The obtained biopsy specimens were then placed on filter paper, and the tissue quantity was macroscopically evaluated. Finally, all specimens were stained in hematoxylin and eosin and immunostained and evaluated by a pathologist.

## Statistical analysis

Fisher’s exact test was used to evaluate differences in the proportions between the two groups. All statistical analyses were conducted using the SPSS 15.0 software package (SPSS Inc., Chicago, IL). *P* values of <0.05 were considered to be statistically significant.

## Results

**Fig. 3:**
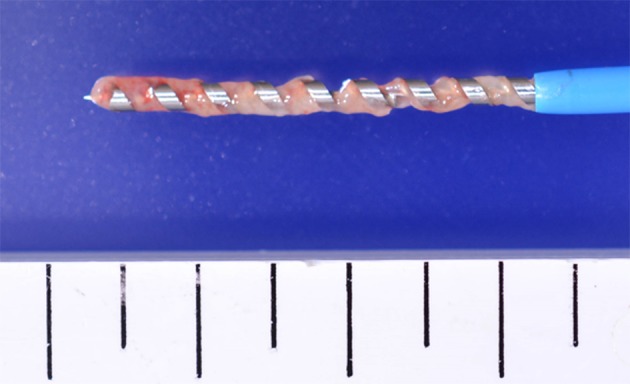
The drill just after withdrawal is shown. The deep helical ditch of the drill is filled with tumor tissue

**Fig. 4:**
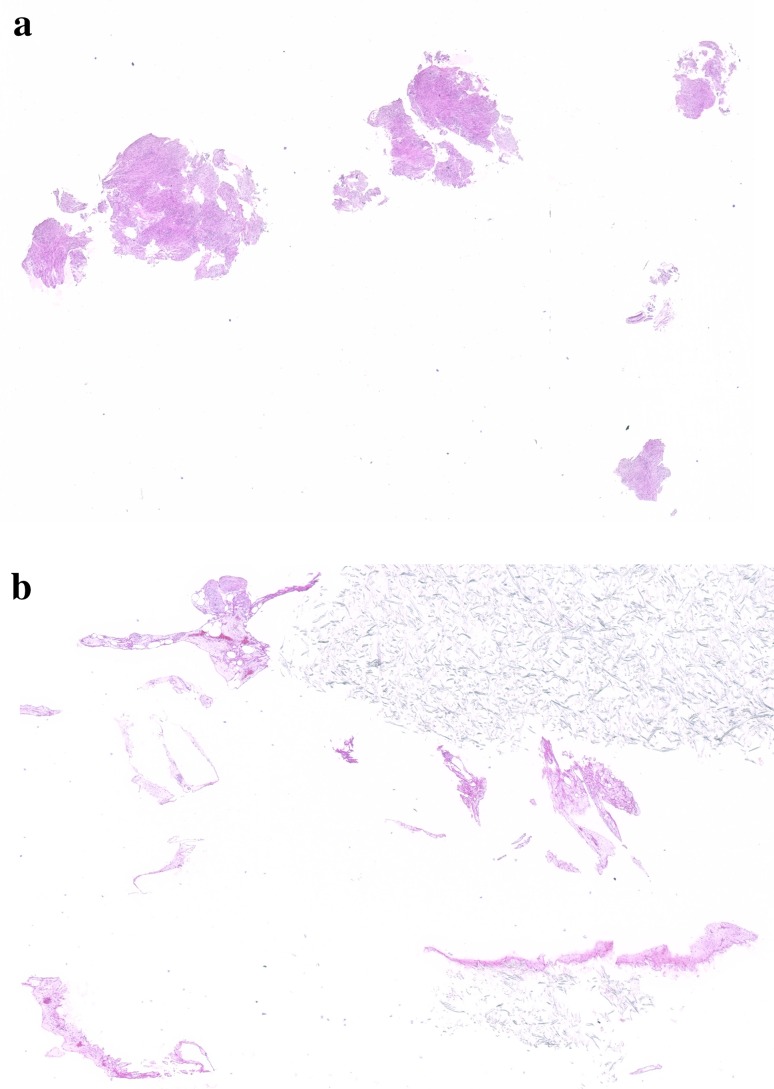
Representative images of specimen no. 1. The tissue obtained using DNAB **a** clearly shows larger clumps than that with FNA **b**, H&E, ×4

The characteristics of the specimens are summarized in Table 1. All 13 drill biopsy specimens were obtained in only one pass each. The drill just after withdrawal is shown in Fig. 3. The deep helical ditch of the drill was filled with tumor tissue. Each specimen obtained by DNAB was macroscopically much greater than that by FNA. For instance, specimens obtained from no. 1 using DNAB clearly showed larger tissue clumps than those obtained using FNA (Fig. 4). The ratio of histologically diagnosed SMT (◎ + ○) was DNAB/FNA = 100 % (13/13)/61.5 % (8/13) (*P* = 0.047). There were significant differences between DNAB and FNA regarding the ratio of microscopic diagnosable tumors <25 mm in the longer axis [100 % (7/7)/42.9 % (3/7), *P* = 0.035]. Moreover, all specimens could be pathologically diagnosed according to the specimens obtained by DNAB. In particular, two gastric SMTs undiagnosed by preoperative EUS-FNA were diagnosed as aberrant pancreas and schwannoma by DNAB. Additionally, a substantial amount of epithelial tissue was obtained from specimen no. 6 using DNAB.

**Table 1:** Comparison of tissue quantity between DNAB and FNA

Specimen (no.)	Tumor diameter (mm)	Diagnostic method	Preoperative diagnosis	Pathological diagnosis by resected specimen	Macroscopic tissue quantity	Microscopic tissue quantity	
						In DNAB	In FNA
1	20 × 15	EUS-FNA	GIST	GIST	DNAB > FNA	◎	△
2	20 × 17 × 16	EUS-FNA	GIST	GIST	DNAB > FNA	○	×
3	22 × 18 × 18	EUS-FNA	GIST	GIST	DNAB > FNA	◎	◎
4	22 × 20 × 20	EUS-FNA	GIST	GIST	DNAB > FNA	◎	◎
5	22 × 20 × 9	EUS-FNA	No tissue	Aberrant pancreas	DNAB > FNA	◎	△
6	25 × 18 × 15	EUS-FNA	GIST	GIST	DNAB > FNA	○	◎
7	25 × 24 × 16	EUS-FNA	No tissue	Schwannoma	DNAB > FNA	◎	×
8	27 × 17 × 10	EUS-FNA	GIST	GIST	DNAB > FNA	◎	○
9	30 × 25 × 20	Bx	GIST	GIST	DNAB > FNA	◎	△
10	35 × 35 × 25	Non-enforcement	Hematoid SMT	GIST	DNAB > FNA	◎	○
11	50 × 40 × 30	EUS-FNA	GIST	GIST	DNAB > FNA	◎	◎
12	60 × 40 × 37	Bx	GIST	GIST	DNAB > FNA	◎	○
13	80 × 60	Bx	Chronic gastritis	GIST	DNAB > FNA	◎	○

*EUS-FNA* endoscopic ultrasound-guided fine-needle aspiration biopsy

*Bx* biopsy

*GIST* gastrointestinal stromal tumor

*SMT* submucosal tumor

*FNA* fine-needle aspiration biopsy on the desk

*DNAB* drill needle aspiration biopsy on the desk

> The left is larger than the right

◎ A sufficient quantity to make a tissue diagnosis

○ An appropriate quantity to make a tissue diagnosis

△ A moderately insufficient quantity to make a tissue diagnosis

× No tissue

## Discussion

DNAB is a novel method that can obtain more tissue than FNA in small gastric SMTs. The main direction of the movement in DNAB is a turn, whereas that in FNA is a back-and-forth movement (so-called stroke). Therefore, DNAB will enable tissue sampling in small tumors having difficulty with tissue sampling in FNA.

GIST was first described in 1983 as a tumor in the gastrointestinal tract and mesentery, characterized by a specific histological and immunohistochemical pattern [1]. Because GIST is considered to be potentially malignant, all GISTs may need to be resected, even small lesions [2]. The European Society for Medical Oncology (ESMO) and the Japanese GIST guidelines recently recommended surgical resection when SMT is diagnosed as an immunohistologically confirmed GIST, even if <2 cm [5–7]. Therefore, a preoperative pathological diagnosis of all gastric SMTs should be obtained. However, even when a biopsy is performed during conventional endoscopy, the GIST is typically covered by normal mucosa, leading to insufficient endoscopic biopsy specimens from deeper layers. In such cases, EUS-FNA is considered to be a reliable and accurate method. EUS-FNA in pancreatic disease was first reported in 1992 [8]. Recently, EUS-FNA has been widely used as a minimally invasive technique that allows identification and sampling of various SMTs and extraintestinal mass lesions [3, 4, 9, 10]. It is very important to obtain adequate tissue for the correct diagnosis in immunohistochemical studies [1]. The collection rate of adequate specimens from SMTs was 74.5–83.9 % [4, 9–11]. In particular, the diagnostic rate of tumors measuring <2 cm in diameter was poor [3, 4]. Regarding reports of good results, the diagnostic rate (first session only) was 62 % in gastric SMTs measuring 1–2 cm [12]. In tumors <2 cm, the distance for stroking within the lesion was too short. Smaller tumors are not more stable to puncture. To solve this problem, the size of the FNA needle or the number of needle passes might not influence the diagnostic yield [3]. There are alternative methods to obtain tissue [13–16]. A Tru-Cut biopsy is not superior to EUS-FNA in GISTs because of the high rate of technical failure of the Tru-Cut device [13, 14]. A bloc biopsy using a mucosal flap method or a keyhole biopsy is an excellent technique for tumors that grow toward the lumen from the muscularis propria [15, 16]. However, these methods cannot be applied for tumors that grow toward the abdominal cavity from the muscularis propria. Akahoshi et al. described that further technical improvement and refinement of devices, including needles and echoendoscopes, are needed to solve such problems [12]. Therefore, we have invented a new device referred to as a drill needle aspiration biopsy (DNAB). This instrument allows clinicians to readily obtain an adequate amount of tumor, safely and accurately, for immunohistological studies. Our drill consists of three main characteristics: a sharp tip, front-like cutter and deep helical ditch. The sharp tip fixes the tumor, the front-like cutter carves the tumor into a spiral, and the deep helical ditch collects the chopped tissue. Furthermore, this new device can be manually turned and aspirated. A pneumatic [17–19] or electric [20] high-speed drill had been used to obtain soft tissue from thyroid, breast, salivary glands and enlarged lymph nodes. Morrison et al. reported better biopsies can be obtained in soft tissue if the speed is further increased using a higher gas pressure [17]. However, those procedures are not performed likely because they are complicated. We were able to perform a simple manual procedure by making the front like a cutter. Moreover, it is said that a gentle negative pressure is necessary, especially in soft tissue, while withdrawing the needle [17]. Therefore, we provided a continuous suction mechanism in DNAB, which is also widely used in FNA.

In our study, there was a difference in the thickness of the needle between DNAB and FNA; however, each specimen obtained by DNAB was macroscopically larger in quantity than that by FNA. Additionally, FNA required more stroke times than DNAB (10 vs. 1). FNA generally requires 3–5 passes and 10–20 stroke times per one pass within the lesion [21]. For the future application of DNAB in the stomach, we believe that having only one stroke time will reduce the risk of making a false pass.

There were significant differences in the ratio of microscopic diagnosable tumors <25 mm in the longer axis between DNAB and FNA. The differences might be associated with the mechanisms of DNAB and FNA. The mechanism of FNA is to plane the tissue by to-and-fro movement and then aspirate it. It is difficult to move the needle in the lesion if the target lesion is small. On the other hand, the mechanism of DNAB is to cut the tissue by turning and then aspirate it.

DNAB was able to obtain a more substantial amount of useable tissue compared with FNA, except in one case that included epithelial tissue. We were surprised that all the tissue obtained by DNAB was useful for a diagnosis. Two gastric SMTs undiagnosed by preoperative EUS-FNA could be diagnosed as aberrant pancreas and schwannoma by DNAB. Therefore, DNAB, with its cutting and vacuuming mechanism, is useful if the tissue is hard.

**Fig. 5:**
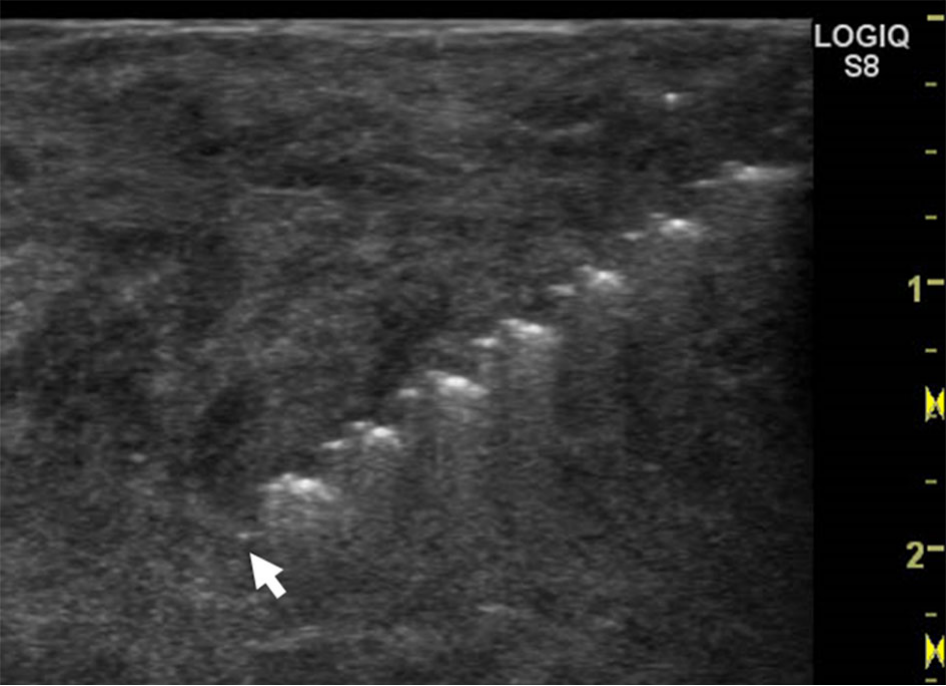
During this pass of DNAB, the drill tip is visualized using ultrasonography. The tip (*arrowhead*) and helical ditch of the drill are clearly observed

In one case, we attempted to watch the drill tip during DNAB using ultrasonography. The drill tip and helical ditch were clearly visualized (Fig. 5). We speculate that the clear depiction of the needle can facilitate the pass in a smaller lesion and provide a safe entry site for the biopsy needle. However, it is necessary to compare the tissue volume between procedures objectively and to decrease the needle diameter. Furthermore, we may approach a deep tumor by exchanging a sheath with a hollow needle to exclude the epithelial content.

In conclusion, we devised a new mechanism called DNAB to obtain more tissue than FNA for small gastric SMTs. However, many challenges remain before it can be used in clinical practice. Future animal experiments to evaluate DNAB under ultrasonography as a pre-stage of EUS-DNAB are currently planned.

## Abbreviations

SMT: Submucosal tumor
DNAB: Drill needle aspiration biopsy
GIST: Gastrointestinal stromal tumor
EUS-FNA: Endoscopic ultrasound-guided fine-needle aspiration biopsy

## References

[1] Blay JY, Bonvalot S, Casali P, Choi H, Debiec-Richter M, Dei Tos AP (2005). Consensus meeting for the management of gastrointestinal stromal tumors. Report of the GIST Consensus Conference of 20–21 March 2004, under the auspices of ESMO. Ann Oncol.

[2] Miettinen M, Sobin LH, Lasota J (2005). Gastrointestinal stromal tumors of the stomach: a clinicopathologic, immunohistochemical, and molecular genetic study of 1765 cases with long-term follow-up. Am J Surg Pathol.

[3] Watson RR, Binmoeller KF, Hamerski CM, Shergill AK, Shaw RE, Jaffee IM (2011). Yield and performance characteristics of endoscopic ultrasound-guided fine needle aspiration for diagnosing upper GI tract stromal tumors. Dig Dis Sci.

[4] Akahoshi K, Sumida Y, Matsui N, Oya M, Akinaga R, Kubokawa M (2007). Preoperative diagnosis of gastrointestinal stromal tumor by endoscopic ultrasound-guided fine needle aspiration. World J Gastroenterol.

[5] Nishida T, Kawai N, Yamaguchi S, Nishida Y (2013). Submucosal tumors: comprehensive guide for the diagnosis and therapy of gastrointestinal submucosal tumors. Dig Endosc.

[6] Nishida T, Hirota S, Yanagisawa A, Sugino Y, Minami M, Yamamura Y (2008). Clinical practice guidelines for gastrointestinal stromal tumor (GIST) in Japan: English version. Int J Clin Oncol.

[7] Koo DH, Ryu MH, Kim KM, Yang HK, Sawaki A, Hirota S, et al. Asian Consensus Guidelines for the Diagnosis and Management of Gastrointestinal Stromal Tumor. Cancer Res Treat. 2016. 10.4143/crt.2016.187 PMC5080813 27384163

[8] Vilmann P, Jacobsen GK, Henriksen FW, Hancke S (1992). Endoscopic ultrasonography with guided fine needle aspiration biopsy in pancreatic disease. Gastrointest Endosc.

[9] Suzuki T, Arai M, Matsumura T, Arai E, Hata S, Maruoka D, et al. Factors Associated with Inadequate Tissue Yield in EUS-FNA for Gastric SMT. ISRN Gastroenterol. 2011;619128. 10.5402/2011/619128 PMC3168491 21991522

[10] Mekky MA, Yamao K, Sawaki A, Mizuno N, Hara K, Nafeh MA (2010). Diagnostic utility of EUS-guided FNA in patients with gastric submucosal tumors. Gastrointest Endosc.

[11] Hoda KM, Rodriguez SA, Faigel DO (2009). EUS-guided sampling of suspected GI stromal tumors. Gastrointest Endosc.

[12] Akahoshi K, Oya M, Koga T, Koga H, Motomura Y, Kubokawa M (2014). Clinical usefulness of endoscopic ultrasound-guided fine needle aspiration for gastric subepithelial lesions smaller than 2 cm. J Gastrointest Liver Dis.

[13] Fernandez-Esparrach G, Sendino O, Sole M, Pellise M, Colomo L, Pardo A (2010). Endoscopic ultrasound-guided fine-needle aspiration and trucut biopsy in the diagnosis of gastric stromal tumors: a randomized crossover study. Endoscopy.

[14] Polkowski M, Gerke W, Jarosz D, Nasierowska-Guttmejer A, Rutkowski P, Nowecki ZI (2009). Diagnostic yield and safety of endoscopic ultrasound-guided trucut [corrected] biopsy in patients with gastric submucosal tumors: a prospective study. Endoscopy.

[15] Kobara H, Mori H, Fujihara S, Nishiyama N, Kobayashi M, Kamata H (2013). Bloc biopsy by using submucosal endoscopy with a mucosal flap method for gastric subepithelial tumor tissue sampling (with video). Gastrointest Endosc.

[16] Grubel P (2010). Keyhole biopsy: an easy and better alternative to fine-needle aspiration or Tru-cut biopsy of submucosal gastrointestinal tumors. Endoscopy.

[17] Morrison R, Deeley TJ (1955). Drill biopsy: a technique using a high-speed drill. J Fac Radiol.

[18] Meyerowitz BR, Roberts T, Volk H (1965). Pneumatic Drill for Tissue Biopsy. Am J Surg.

[19] Sachdeva HS, Wig JD, Dutta BN (1973). Evaluation of high speed pneumatic drill biopsy in diagnosis of tumours. Indian J Cancer.

[20] Samejima N, Nakajima S (1976). New electric drill biopsy apparatus for breast tumors. Surgery.

[21] Irisawa A, Hikichi T, Bhutani MS, Ohira H (2009). Basic technique of FNA. Gastrointest Endosc.

